# Job satisfaction of foreign-national physicians working in patient care: a cross-sectional study in Saxony, Germany

**DOI:** 10.1186/s12995-016-0129-2

**Published:** 2016-08-30

**Authors:** Birte Pantenburg, Katharina Kitze, Melanie Luppa, Hans-Helmut König, Steffi G. Riedel-Heller

**Affiliations:** 1 Institute of Social Medicine, Occupational Health and Public Health, University of Leipzig, Philipp-Rosenthal-Str. 55, 04103 Leipzig, Germany; 2 State Office of Tax and Finance (Landesamt für Steuern und Finanzen), Occupational Health Management, Stauffenbergallee 2, 01099 Dresden, Germany; 3 Department of Health Economics and Health Services Research, University Medical Center Hamburg-Eppendorf, Martinistr. 52, 20246 Hamburg, Germany

**Keywords:** Physicians, Job satisfaction, Migration, Germany

## Abstract

**Background:**

Physician migration is gaining attention worldwide. Despite increasing numbers of foreign physicians in Germany, their perceptions on working in Germany remain unexplored. Within a large survey on Saxon physicians, the aim of this study was to elucidate whether foreign-national physicians’ job satisfaction differed from German physicians’ job satisfaction.

**Methods:**

The study was designed as a comprehensive cross-sectional survey. All physicians ≤40 years and registered with the State Chamber of Physicians of Saxony (*n* = 5956) were mailed a paper-pencil questionnaire, of which 2357 were returned (response rate = 40 %). Questionnaires addressed socio-demographics and assessed job satisfaction by asking participants to rate their satisfaction with the overall job situation and 20 different aspects on a 5-point Likert scale (1 = very dissatisfied to 5 = very satisfied).

**Results:**

Ten percent of participants were foreign-national physicians. The three main countries of origin were the Czech Republic, Slovakia, and Poland. Foreign-national physicians were more satisfied with aspects related to patient care, such as “possibility to treat patients as you deem optimal” and “relationship with patients”. However, they were less satisfied with aspects related to human relations, such as “work atmosphere”, relationship with co-workers, and “social status”. Foreign-national physicians were also less satisfied with the aspect “work enjoyment”.

**Conclusions:**

Further research on determinants promoting foreign-national physicians’ job satisfaction is needed as their professional well-being may influence quality of patient care. Measures teaching cross-cultural competence and awareness may be beneficial for both foreign-national and German physicians.

**Electronic supplementary material:**

The online version of this article (doi:10.1186/s12995-016-0129-2) contains supplementary material, which is available to authorized users.

## Introduction

Global physician migration has been gaining increasing attention [1]. The number of foreign physicians working in Germany has risen from 16,818 in 2007 to 21,650 in 2010, and to 34,706 in 2014 [2]. Vacancies, primarily in rural areas and in eastern Germany, are being filled with immigrant physicians [3]. The State Chamber of Physicians in Saxony underlines foreign physicians’ important contributions to the quality of patient care and to safeguarding the operability of hospital wards, especially in rural areas [4]. Thus, foreign physicians play an increasingly important role in the German health care system, above all in the hospital sector [5].

While it has been shown that physicians’ professional satisfaction seems to be positively associated with their patients’ satisfaction with care [6], data on immigrant physicians’ perspectives on their work in Germany are scarce to non-existent. Therefore, in the context of a larger survey on Saxon physicians’ job satisfaction [7], burnout [8], and wishes to quit [9], the aim of the present analysis was to elucidate whether foreign-national physicians’ job satisfaction differed from German physicians’ job satisfaction.

## Methods

### Study population and study design

In a cross-sectional survey all physicians ≤ 40 years and registered with the State Chamber of Physicians of Saxony (*n* = 5956) were sent a paper-pencil questionnaire, accompanied by information about the study’s purpose, anonymous design, and that returning the completed questionnaire would be regarded as giving informed consent to participation. From September 2012 to February 2013, 2357 questionnaires were returned (response rate = 40 %). The ethics committee of the University of Leipzig (Ethik-Kommission an der Medizinischen Fakultät der Universität Leipzig) approved the study.

### Questionnaire and data analysis

The questionnaire was developed based on a literature search and on existing instruments as described elsewhere [8]. Briefly, it included questions on socio-demographics, as well as job satisfaction (mainly based on [10], modified considering [11] and [12]), asking participants to rate their satisfaction with the overall job situation and 20 different aspects on a 5-point Likert scale (1 = very dissatisfied to 5 = very satisfied).

Of the 2357 questionnaires returned, 9 were excluded because they either did not meet the inclusion criteria or were incomplete. The present analysis included *n* = 1893 physicians working in patient care who provided an answer (yes/no) to the question “Do you have German citizenship?”. Chi^2^-tests and t-tests were used to compare sociodemographic characteristics of participants with and without German citizenship. Means were calculated for each item of the job satisfaction questionnaire and comparisons were performed using t-tests. Data was analysed with Stata 13.0 for Windows.

## Results

### Characteristics of the study population

The characteristics of the study population are detailed in Table 1. Ten percent (192/1893) of the study population reported a citizenship other than German (Table 2).

**Table 1:** Characteristics of the study population

	Citizenship		
	German (*n* = 1701)	Other (*n* = 192)	*p*
	% (*n*)	% (*n*)	
Women	61.3 (1043)	62.0 (119)	0.858
In relationship	85.2 (1446)	71.9 (138)	<0.001
Has children	54.5 (927)	36.7 (70)	<0.001
Specialist	38.0 (645)	34.4 (66)	0.325
Full-time	83.0 (1411)	93.8 (180)	<0.001
Inpatient setting	82.5 (1377)	92.1 (174)	0.001
Urban setting	77.4 (1261)	69.9 (123)	0.026
Leading position/own practice	15.2 (253)	6.9 (13)	0.002
	M (SD; 95 % CI)	M (SD; 95 % CI)	
Age (years)	32.92 (3.95; 32.73–33.11)	32.09 (4.15; 31.50–32.68)	0.006
Work experience (years)	5.51 (3.75; 5.33–5.69)	5.59 (3.90; 5.03–6.14)	0.785

Results of participants with and without German citizenship were compared and *p* values are shown. Percentages were calculated with the number of answers actually given. *M* mean, *SD* standard deviation, *CI* confidence interval

**Table 2:** Country of citizenship of foreign-national physicians

Country	% (*n*)
Czech Republic	16.1 (31)
Slovakia	13.5 (26)
Poland	10.4 (20)
Austria	9.4 (18)
Romania	8.9 (17)
Hungary	4.2 (8)
Ukraine	3.1 (6)
Syria	2.6 (5)
Bulgaria	2.1 (4)
Greece	2.1 (4)
Russia	2.1 (4)
Lithuania	1.6 (3)
Bosnia	1.0 (2)
France	1.0 (2)
Italy	1.0 (2)
Moldova	1.0 (2)
Azerbaijan	0.5 (1)
Belarus	0.5 (1)
Georgian Republic	0.5 (1)
India	0.5 (1)
Indonesia	0.5 (1)
Kosovo	0.5 (1)
Latvia	0.5 (1)
Lebanon	0.5 (1)
Luxemburg	0.5 (1)
Macedonia	0.5 (1)
Mexico	0.5 (1)
Serbia	0.5 (1)
Switzerland	0.5 (1)
Turkey	0.5 (1)
Not specified	12.5 (24)
Total	100 (192)

### Work satisfaction of participants with and without German citizenship

Participants without German citizenship were more satisfied with the “possibility to treat patients as you deem optimal”, “relationship with patients”, and “equality of men and women”. They were less satisfied with the “work atmosphere”, “social status”, “relationship with colleagues”, “relationship with non-medical staff”, and “job security”. Foreign-national physicians were also less satisfied with the aspect “work enjoyment” (Table 3).

**Table 3:** Work satisfaction of participants with and without German citizenship

	German citizenship	Other citizenship	*p*
	M (SD; 95 % CI)	M (SD; 95 % CI)	
Overall job situation	3.41 (0.94; 3.36–3.45)	3.39 (0.93; 3.25–3.53)	0.799
Time for administrative tasks	2.25 (1.02; 2.21–2.30)	2.30 (1.04; 2.15–2.45)	0.528
Time for family, friends, leisure activities	2.47 (1.12; 2.42–2.53)	2.35 (1.00; 2.21–2.50)	0.155
Stress level at work	2.71 (1.02; 2.67–2.76)	2.61 (0.94; 2.47–2.74)	0.164
Work load	2.87 (1.10; 2.82–2.92)	2.78 (1.03; 2.63–2.93)	0.265
Possibility to treat patients as you deem optimal	3.18 (0.92; 3.14–3.23)	3.40 (0.93; 3.26–3.53)	0.003
Training opportunities	3.25 (1.06; 3.20–3.30)	3.26 (1.08; 3.11–3.41)	0.889
Career opportunities	3.27 (0.96; 3.23–3.32)	3.36 (0.95; 3.22–3.49)	0.258
Intellectual stimulation at work	3.54 (0.94; 3.50–3.59)	3.47 (0.89; 3.34–3.60)	0.290
Possibility to refer patients to specialists whenever you deem it necessary	3.58 (0.92; 3.54–3.63)	3.72 (0.84; 3.59–3.84)	0.058
Work enjoyment	3.68 (0.91; 3.64–3.72)	3.53 (0.94; 3.39–3.66)	0.028
Quality of the medical care you provide	3.69 (0.71; 3.66–3.72)	3.78 (0.75; 3.67–3.88)	0.111
Income	3.70 (0.95; 3.66–3.75)	3.62 (0.92; 3.49–3.75)	0.251
Equality of women and men	3.71 (1.03; 3.66–3.76)	3.88 (1.02; 3.73–4.02)	0.038
Relationship with superiors	3.75 (1.00; 3.71–3.80)	3.66 (0.97; 3.52–3.80)	0.215
Work atmosphere	3.76 (0.94; 3.72–3.81)	3.54 (0.99; 3.40–3.69)	0.003
Social status	3.77 (0.91; 3.73–3.82)	3.61 (0.97; 3.47–3.75)	0.020
Relationship/professional exchange with colleagues	3.83 (0.87; 3.78–3.87)	3.67 (0.90; 3.55–3.80)	0.023
Relationship with patients	3.94 (0.65; 3.91–3.97)	4.04 (0.76; 3.93–4.14)	0.046
Relationship with non-medical staff	3.96 (0.77; 3.92–3.99)	3.73 (0.86; 3.61–3.85)	<0.001
Job security	4.04 (0.94; 4.00–4.09)	3.79 (0.96; 3.66–3.93)	<0.001

Means of 5-point Likert scales (1 = very dissatisfied to 5 = very satisfied) were calculated. Participants with and without German citizenship were compared and *p* values are shown. *M* mean, *SD* standard deviation, *CI* confidence interval

## Discussion

Of this sample of physicians in Saxony, 10.1 % stated a citizenship other than German. This is consistent with official Saxon statistics according to which 10.5 % of working physicians were foreign [4]. Also, the source countries in this sample mainly follow official statistics, with the three most important countries being the Czech Republic, Slovakia, and Poland [4].

Data from Germany on immigrant physicians’ job satisfaction is extremely limited. In the present study, participants with and without German citizenship did not differ regarding their overall work satisfaction. However, foreign-national participants were more satisfied with aspects related to patient care, such as the “possibility to treat patients as you deem optimal”, and “relationship with patients”. In previous interviews, 20 Polish physicians named better working conditions, and advanced training or qualification possibilities as important reasons for migrating to Germany [13]. Similarly, better working environment, access to specialist training, as well as equipment available in German hospitals, was an important reason for physicians migrating from countries such as Austria, Bulgaria, India, Iraq, Latvia, Mongolia, and Syria [14]. If expectations are met, this may contribute to immigrant physicians’ relatively higher satisfaction with the possibility for optimal treatment and subsequently, their doctor-patient-relationship. Although higher wages have also been identified as a major incentive for migration to Germany [13, 14], there was no difference in satisfaction between participants with and without German citizenship.

Foreign-national physicians were less satisfied with aspects related to human relations, such as “work atmosphere”, “relationship with colleagues”, “relationship with non-medical staff”, and “social status”. An exception was “equality of women and men”, which foreign-national physicians were more satisfied with. Language difficulties may hinder immigrant physicians in Germany from “integrating into the team” and point to cultural barriers immigrant physicians may face when coming to Germany [14]. Immigrant physicians’ professional socialization may differ from that of German physicians, leading to diverging ideas of who is responsible for which professional tasks and decisions [15]. If a foreign-national physician subsequently does not act as expected, German colleagues may interpret this as lack of competence or refusal to work [15]. This, together with language barriers and a high work load, may cause severe conflicts within the team [15]. A study from the USA showed that international medical graduates experienced workplace bias and discrimination [16]. It is conceivable that foreign-national physicians in Germany have similar experiences, subsequently impairing their work enjoyment and their satisfaction with the work atmosphere, relationship with co-workers, and social status. International medical graduates in the USA reported “limitations to professional opportunities” and were less satisfied with their careers than US medical graduates [16, 17]. Again, similar phenomena, alongside difficulties with recognition of foreign degrees and qualifications by German authorities [13, 14], may contribute to foreign-national physicians’ lower satisfaction with job security as well as social status.

Physicians’ professional satisfaction is positively linked to their patients’ satisfaction with care [6]. Similarly, a recent systematic review concludes that physicians’ occupational well-being (including the aspect of job satisfaction) could contribute to quality of patient care in terms of better patient satisfaction, adherence to treatment recommendations, interpersonal aspects of care, and quality of overall care processes [18]. Foreign-national physicians are an important part of the German health care system: e.g. 20.8 % of physicians practising in North Rhine-Westphalia in 2015 had a citizenship other than German (calculations based on [19] and [20]). Therefore, foreign-national physicians’ occupational well-being, including job satisfaction, may influence quality of patient care in Germany.

### Limitations

While citizenship is an indicator of migration, it does not permit differentiating between foreign-born and foreign-trained physicians, nor is it proof of being born in the country of citizenship. It is unknown how long foreign-national physicians had been living in Germany and whether they planned on staying permanently or for a limited time-period. As it was beyond the scope of this survey, the questionnaire did not address language skills. Therefore, it is not possible to evaluate the influence of language skills on job satisfaction in this sample of foreign-national physicians. In the context of the main aim of the study, which was to evaluate young physicians’ wishes to leave patient care [9] and to go abroad for clinical work, physicians up to 40 years of age were invited to participate in this study. Larger, less age-restricted studies are needed to paint a comprehensive picture of foreign-national physicians’ perspectives on their professional situation in Germany. Anonymization prevented characterization of those who refused participation.

## Conclusions

In the present study, foreign-national physicians experienced less work enjoyment and were less satisfied with aspects mainly related to human relations, such as “work atmosphere”, relationship with co-workers, and “social status”. While in this analysis the reasons remain speculative, previous studies point to divergent professional socializations, language and cultural barriers, workplace bias and discrimination as potential explanations [14–16]. There is an urgent need for further research exploring determinants of job satisfaction of foreign-national physicians practicing in Germany as their professional well-being may influence quality of patient care. Potential measures to improve foreign-nationals’ job satisfaction may include extended periods of adjustment when starting work in the German health care system, language classes and courses aiding migrant physicians with understanding and adjusting to the German culture. Importantly, German physicians working with foreign-national colleagues may also benefit from educational measures teaching cross-cultural competence and awareness, in an effort to facilitate effective work in a multi-national team.

## Additional file

Additional file 1: Study questionnaire. (PDF 198 kb)
