# Supplementary material for: Job satisfaction of foreign-national physicians working in patient care: a cross-sectional study in Saxony, Germany
Source: J Occup Med Toxicol. 2016 Aug 30;11(1):41. doi: 10.1186/s12995-016-0129-2 (PMC5006605; doi:10.1186/s12995-016-0129-2)
Supplement: Additional file 1: — Study questionnaire. (PDF 198 kb) [file 12995_2016_129_MOESM1_ESM.pdf]

UNIVERSITÄT LEIPZIG
Medizinische Fakultät

Fragebogen zur

Ausstiegsbereitschaft von Ärztinnen und Ärzten in Sachsen

durchgeführt
unter der Leitung von Frau Prof. Dr. med. Steffi G. Riedel-Heller, MPH
Institut für Sozialmedizin, Arbeitsmedizin und Public Health (ISAP)
Philipp-Rosenthal-Str. 55
04103 Leipzig

Ansprechpartnerin: Frau Dr. med. Birte Pantenburg
Telefon: 0341 - 97 24 564
Email: Birte.Pantenburg@medizin.uni-leipzig.de

Sehr geehrte Frau Kollegin, sehr geehrter Herr Kollege,
herzlichen Dank, dass Sie sich dafür entschieden haben, den Fragebogen auszufüllen! Nicht alle Fragen werden auf Sie zutreffen. Wir bitten Sie deshalb, beim Ausfüllen genau den Anweisungen zu folgen. Hier sind zunächst einige Fragen zu Ihrer Person.

1.	Sind Sie weiblich oder männlich?	☐ 1 Weiblich<br>☐ 2 Männlich
2.	Wie alt sind Sie?	_______Jahre
3.	Sie leben derzeit	☐ 1 Alleine<br>☐ 2 In einer Partnerschaft
4.	Haben Sie Kinder, die in Ihrem Haushalt leben?	☐ 0 Nein ⇨ **weiter mit Frage 6**<br>☐ 1 Ja ⇨ **weiter mit Frage 5**
5.	Wenn ja, was ist das Alter der Kinder/des Kindes?	_____ , _____, _____, _____ Jahr(e)
6.	Haben Sie die deutsche Staatsbürgerschaft?	☐ 1 Ja<br>☐ 0 Nein und zwar (bitte nennen):<br>___________________________________

7.	Haben Sie schon einmal 3 Monate oder länger außerhalb Deutschlands verbracht? (Mehrfachnennungen möglich)	
☐ 0	Nein	☐ 2 Ja, zur klinischen Tätigkeit
☐ 1	Ja, zum Medizinstudium	☐ 3 Ja, zum Forschen
☐ 99	Sonstiger Grund (bitte nennen):<br>___________________________________	

Bitte beantworten Sie nun die folgenden Fragen zu Ihrer beruflichen Situation.

8.	Verfügen Sie über eine Approbation oder eine Berufserlaubnis?	☐ 1 Approbation<br>☐ 2 Berufserlaubnis
9.	Seit wann?	___________ (Jahreszahl)
10.	Machen Sie zurzeit ein Aufbaustudium, bzw. haben Sie ein Aufbaustudium abgeschlossen (z.B. Master of Public Health, Master of Business Administration?)	☐ 0 Nein<br>☐ 1 Ja und zwar (bitte **nicht** abkürzen!):<br>___________________________________

11.	Über wie viele Jahre Berufserfahrung in der klinischen Tätigkeit verfügen Sie?	☐ 0 Ich habe keine klinische Erfahrung<br>☐ 1 Meine klinische Erfahrung beträgt<br>_________________________ Jahr(e)

12.	Sind Sie Fachärztin/Facharzt?	☐ 0 Nein<br>☐ 1 Ja

13. Sind Sie **Fachärztin/arzt**, geben Sie bitte an, welche Qualifikation(en) Sie **erworben haben**. Sind Sie **kein(e) Fachärztin/arzt**, geben Sie bitte an, welche Qualifikation(en) Sie **erwerben möchten** (Mehrfachnennungen möglich).

☐ 0	Keine	☐ 13	Mikrobiologie, Virologie und Infektionsepidemiologie
☐ 1	Allgemeinmedizin	☐ 14	Neurochirurgie
☐ 2	Anästhesie	☐ 15	Neurologie
☐ 3	Arbeitsmedizin	☐ 16	Nuklearmedizin
☐ 4	Augenheilkunde	☐ 17	Orthopädie
☐ 5	Chirurgie	☐ 18	Pädiatrie
☐ 6	Dermatologie	☐ 19	Pathologie
☐ 7	Gynäkologie	☐ 20	Psychiatrie und Psychotherapie
☐ 8	HNO	☐ 21	Psychosomatische Medizin und Psychotherapie
☐ 9	Innere Medizin	☐ 22	Radiologie
☐ 10	Dermatologie	☐ 23	Strahlentherapie
☐ 11	Kinder- und Jugendpsychiatrie	☐ 24	Transfusionsmedizin
☐ 12	Labormedizin	☐ 25	Urologie

☐ 99 Sonstige Facharztqualifikation (bitte nennen):

____

14.	Würden Sie sich noch einmal dafür entscheiden Ärztin/Arzt zu werden?	☐ 0 Nein<br>☐ 1 Ja<br>☐ 2 Weiß nicht

15.	Sind Sie zurzeit berufstätig?<br>(wenn Sie sich zurzeit in Elternzeit befinden, kreuzen Sie bitte Nein an)	☐ 0 Nein ⇨ **weiter mit Frage 32, Seite 9**<br>☐ 1 Ja ⇨ **weiter mit Frage 16, Seite 3**

Wir möchten in den folgenden Fragen gerne von Ihnen wissen, wie zufrieden Sie mit Ihrer derzeitigen beruflichen Situation sind.

16. Bitte geben Sie an, zu welchem Grad Sie derzeit **insgesamt** mit Ihrer Berufssituation zufrieden sind.

Äußerst unzufrieden	Eher unzufrieden	Teils, teils	Eher zufrieden	Äußerst zufrieden
①	②	③	④	⑤

17. Bitte geben Sie nun an, zu welchem Grad Sie mit den folgenden **Aspekten** Ihres Berufslebens zufrieden sind:

	Äußerst unzufrieden	Eher unzufrieden	Teils, teils	Eher zufrieden	Äußerst zufrieden
Ihre Arbeitsbelastung	①	②	③	④	⑤
Ihre intellektuelle Anregung bei der Arbeit	①	②	③	④	⑤
Die Zeit, die Sie Ihrer Familie, Ihren Freunden oder Ihrer Freizeitgestaltung widmen können	①	②	③	④	⑤
Das Stressniveau, dem Sie bei Ihrer Berufsausübung ausgesetzt sind	①	②	③	④	⑤
Ihr Verhältnis zu Ihren Vorgesetzten	①	②	③	④	⑤
Ihr Verhältnis zu und den beruflichen Meinungsaustausch mit anderen Kolleginnen und Kollegen	①	②	③	④	⑤
Die für administrative Aufgaben aufgewendete Zeit und Energie	①	②	③	④	⑤
Ihre Fortbildungsmöglichkeiten	①	②	③	④	⑤
Ihre Karrierechancen	①	②	③	④	⑤
Ihre Freude an der Arbeit	①	②	③	④	⑤
Das Arbeitsklima	①	②	③	④	⑤
Ihre gesellschaftliche Stellung und der Ihnen entgegengebrachte Respekt	①	②	③	④	⑤
Ihr gegenwärtiges Einkommen	①	②	③	④	⑤
Die Sicherheit Ihres Arbeitsplatzes	①	②	③	④	⑤
Gleichberechtigung von Frauen und Männern	①	②	③	④	⑤

18. Sind Sie zum Zeitpunkt dieser Befragung **hauptsächlich klinisch** tätig?

☐ 1	Ja, ich arbeite zurzeit in Vollzeit	⇨ **weiter mit Frage 19, Seite 4**
☐ 2	Ja, ich arbeite zurzeit in Teilzeit	⇨ **weiter mit Frage 19, Seite 4**
☐ 3	Nein, ich arbeite zurzeit in einer nicht-klinischen Tätigkeit (Vollzeit oder Teilzeit)	⇨ **weiter mit Frage 35, Seite 10**

Sie sind derzeit **hauptsächlich klinisch** tätig. Wir möchten Sie deshalb bitten, auch die folgenden Fragen zur Zufriedenheit mit Ihrer beruflichen Situation zu beantworten.

19. Bitte geben Sie an, zu welchem Grad Sie mit den folgenden **Aspekten** Ihres Berufslebens zufrieden sind:					
	**Äußerst unzufrieden**	**Eher unzufrieden**	**Teils, teils**	**Eher zufrieden**	**Äußerst zufrieden**
Ihr Verhältnis zu Ihren nicht-ärztlichen Mitarbeitern (Pflegekräfte, Arzthelfer/in usw.)	①	②	③	④	⑤
Ihr Verhältnis zu Ihren Patienten	①	②	③	④	⑤
Ihre Möglichkeit, Ihre Patienten so zu behandeln, wie Sie es für optimal erachten	①	②	③	④	⑤
Ihre Möglichkeit, Ihre Patienten an Spezialisten zu überweisen, wann immer Sie es für notwendig halten	①	②	③	④	⑤
Die Qualität der von Ihnen erbrachten medizinischen Versorgung	①	②	③	④	⑤

Nun möchten wir gerne von Ihnen wissen, wie Sie derzeit Ihre Arbeit empfinden.

20.	***Maslach Burnout Inventar aus Copyright-Gründen entfernt***

Bitte beantworten Sie auch die folgenden Fragen zu Ihrer beruflichen Situation.

21. Welche Position haben Sie zurzeit inne?			
**Eher stationärer Bereich**		**Eher ambulanter Bereich**	
☐ 1	Chefärztin/arzt	☐ 5	Ärztin/Arzt in eigener Niederlassung mit GKV*-Vertragsarzttätigkeit
☐ 2	Leitende(r) Oberärztin/arzt	☐ 6	Ärztin/Arzt in eigener Niederlassung ohne GKV*-Vertragsarzttätigkeit
☐ 3	Oberärztin/arzt	☐ 7	Angestellte(r) Ärztin/Arzt in Praxis
☐ 4	Stationsärztin/arzt	☐ 8	Angestellte(r) Ärztin/Arzt in Medizinischem Versorgungszentrum
☐ 88	Sonstiges (bitte nennen): ________________________________	☐ 99	Sonstiges (bitte nennen): ________________________________

*gesetzliche Krankenversicherung

➥ **Bitte weiter mit Frage 22** ➥ **Bitte weiter mit Frage 23**

22.	Sie arbeiten in einer Klinik mit insgesamt	☐ 1	Mehr als 100 Betten
		☐ 2	Weniger als 100 Betten

➥ **Bitte weiter mit Frage 24**

23.	Sie sind	☐ 1	Hausärztlich tätig
		☐ 2	Fachärztlich tätig

➥ **Bitte weiter mit Frage 24**

24.	Wo praktizieren Sie?	☐ 1	Eher im städtischen Bereich
		☐ 2	Eher im ländlichen Bereich

➥ **Bitte umblättern und weiter mit nächster Frage (Frage 25, Seite 7)**

In Deutschland wird derzeit intensiv darüber debattiert, ob und warum immer mehr Ärztinnen und Ärzte **zur klinischen Tätigkeit** ins Ausland abwandern. Wir würden gerne von Ihnen wissen, ob auch Sie sich schon einmal mit dem Gedanken getragen haben, zur klinischen Tätigkeit ins Ausland zu gehen (bitte beachten Sie, dass es nur um **klinische Tätigkeit** im Ausland geht, **nicht** aber um **nicht-klinische** Tätigkeit, wie z.B. ein Forschungsaufenthalt).

25.	Wie sehr würden Sie sich derzeit **wünschen**, zur klinischen Tätigkeit ins Ausland gehen zu können? Bitte kreuzen Sie die Skala entsprechend an.			
**Überhaupt nicht**	**Eher nein**	**Weiß nicht**	**Eher ja**	**Unbedingt**
①	②	③	④	⑤

26.	Haben Sie sich schon einmal eingehend damit auseinandergesetzt, welche Möglichkeiten es für Sie gäbe, im Ausland zu praktizieren?	☐ 0	Nein
		☐ 1	Ja, vor mehr als 3 Jahren
		☐ 2	Ja, in den letzten 3 Jahren

27. Für wie **wahrscheinlich** halten Sie es, dass Sie in den nächsten 5 Jahren zur klinischen Tätigkeit ins Ausland gehen werden….					
	**Ausge-schlossen**	**Eher nein**	**Unent-schieden**	**Eher ja**	**Ganz bestimmt**
…für eine **befristete** Zeit	①	②	③	④	⑤
…für eine **unbefristete** Zeit	①	②	③	④	⑤

28.	Welches Land, außer Deutschland, könnten Sie sich **am ehesten** vorstellen, um klinisch tätig zu sein (bitte **nur ein** Land ankreuzen)?		
☐ 0	Keines	☐ 4	Österreich
☐ 1	Australien	☐ 5	Schweden
☐ 2	Frankreich	☐ 6	Schweiz
☐ 3	Großbritannien	☐ 7	USA
☐ 99	Anderes Land (bitte nennen): ______________________________		

In Deutschland herrscht momentan auch eine angeregte Diskussion darüber, ob und warum immer mehr Ärztinnen und Ärzte **aus der klinischen Tätigkeit ausscheiden**. Wir würden gerne von Ihnen wissen, ob auch Sie sich schon einmal mit dem Gedanken getragen haben, die klinische Tätigkeit aufzugeben.

29.	Wie sehr würden Sie sich derzeit **wünschen**, aus der klinischen Tätigkeit auszusteigen? Bitte kreuzen Sie die Skala entsprechend an.			
**Überhaupt nicht**	**Eher nein**	**Weiß nicht**	**Eher ja**	**Unbedingt**
①	②	③	④	⑤

30.	Haben Sie sich schon einmal eingehend damit auseinandergesetzt, welche beruflichen Alternativen zur klinischen Tätigkeit es für Sie gäbe?	
	☐ 0	Nein
	☐ 1	Ja, vor mehr als 3 Jahren
	☐ 2	Ja, in den letzten 3 Jahren

31. Für wie **wahrscheinlich** halten Sie es, dass Sie in den nächsten 5 Jahren aus der klinischen Tätigkeit aussteigen werden…					
	**Ausge-schlossen**	**Eher nein**	**Unent-schieden**	**Eher ja**	**Ganz bestimmt**
…für eine **befristete** Zeit aus **persönlichen** Gründen (z.B. Elternzeit, Pflege Angehöriger)	①	②	③	④	⑤
…für eine **befristete** Zeit aus **beruflichen** Gründen (z.B. Forschungs-/ Lehrtätigkeit, berufliche Neuorientierung)	①	②	③	④	⑤
…für eine **unbefristete** Zeit aus **persönlichen** Gründen (z.B. Elternzeit, Pflege Angehöriger)	①	②	③	④	⑤
…für eine **unbefristete** Zeit aus **beruflichen** Gründen (z.B. Forschungs-/ Lehrtätigkeit, berufliche Neuorientierung)	①	②	③	④	⑤

↪ **Bitte weiter mit Frage 37, Seite 11**

Sie sind derzeit **nicht berufstätig**. Bitte nennen Sie uns die Gründe dafür.

32.	Warum sind Sie derzeit **nicht berufstätig**?
☐ 1	Ich betreue mein(e) Kind(er)
☐ 2	Ich pflege andere Angehörige
☐ 3	Ich bin arbeitssuchend / arbeitslos
☐ 99	Sonstiger Grund (bitte nennen): ______________________

33.	Waren Sie zuletzt klinisch oder nicht-klinisch tätig?	
☐ 1	Ich war zuletzt klinisch tätig	⇨ **bitte umblättern und weiter mit Frage 35, Seite 10**
☐ 2	Ich war zuletzt nicht-klinisch tätig	⇨ **bitte umblättern und weiter mit Frage 35, Seite 10**
☐ 0	Ich war noch nie berufstätig	⇨ **bitte weiter mit Frage 34**

34.	Möchten Sie in Zukunft eher klinisch oder eher nicht-klinisch tätig sein?	☐ 1 Eher klinisch<br>☐ 2 Eher nicht-klinisch<br>☐ 3 Weiß nicht

↪ Wenn Sie **noch nie berufstätig** waren, **beenden** Sie den Fragebogen bitte hier. Herzlichen Dank für Ihre Mitarbeit!

Sie sind derzeit **nicht-klinisch tätig** oder **nicht berufstätig**.
Bitte beantworten Sie die folgenden Fragen.

35.	Wie lange sind Sie **nicht** mehr **klinisch tätig**, bzw. wie lange haben Sie Ihre klinische Tätigkeit **unterbrochen**?	☐ 0 Ich war noch nicht klinisch tätig<br>______________ Jahr(e)

36.	Haben Sie Ihre Auszeit von, bzw. das Nichtaufnehmen der klinischen Tätigkeit von vornherein zeitlich begrenzt?
☐ 0	Nein
☐ 1	Ja, nicht länger als 12 Monate
☐ 2	Ja, mehr als 12 Monate bis 3 Jahre
☐ 3	Ja, mehr als 3 Jahre

➥ **Bitte umblättern und weiter mit nächster Frage (Frage 37, Seite 11)**

Im Folgenden nennen wir Ihnen mögliche Gründe, sich gegen eine klinische Tätigkeit zu entscheiden.

37.	Wenn Sie **derzeit klinisch tätig** sind, denken Sie bitte an Ihre **jetzige** Situation. Wenn Sie **derzeit nicht-klinisch** tätig, bzw. **nicht berufstätig** sind, denken Sie bitte an Ihre **letzte** klinische Tätigkeit. Bitte kreuzen Sie an, ob der jeweilige Umstand auf Sie zutrifft (zutraf). Bitte kreuzen Sie dann an, welche der auf Sie zutreffenden Gründe für Sie die **3 wichtigsten** für einen Ausstieg aus der klinischen Tätigkeit waren (wären).<br>Waren Sie **nie klinisch tätig**, kreuzen Sie bitte an, welches die **3 wichtigsten** Gründe dafür waren, dass Sie sich gegen die Aufnahme einer klinischen Tätigkeit entschieden haben.

	**Trifft nicht auf mich zu**	**Trifft auf mich zu**	**Drei wichtigste Gründe**
Schlechte Vereinbarung von Beruf und Familie wegen langen/unregelmäßigen Arbeitszeiten	⓪	①	○
Schlechte Vereinbarung von Beruf und Familie wegen unzureichenden Kinderbetreuungsmöglichkeiten	⓪	①	○
Schlechte Vereinbarung von Beruf und privaten Interessen (Hobbys, Freunde etc.)	⓪	①	○
Verhinderung der Ausübung der klinischen Tätigkeit aus gesundheitlichen Gründen	⓪	①	○
Hohe Belastung durch Bereitschafts- und/oder Schichtdienste	⓪	①	○
Fehlende Möglichkeiten zur flexiblen Arbeitszeitgestaltung (z.B. Teilzeitangebote)	⓪	①	○
Regelmäßige Überstunden	⓪	①	○
Hohe Arbeitsbelastung	⓪	①	○
Schlechtes Arbeitsklima	⓪	①	○
Wenig Kollegialität	⓪	①	○
Mobbing	⓪	①	○
Konflikte/Schwierigkeiten mit Vorgesetzten	⓪	①	○
Unzufriedenheit mit Führungsstil meiner/meines Vorgesetzten	⓪	①	○
Fehlende Karrierechancen	⓪	①	○
Unangemessene Bezahlung	⓪	①	○
Hoher Umfang an Verwaltungsarbeit	⓪	①	○
Wenig Zeit für Patientinnen und Patienten	⓪	①	○
Formale Gründe (Ablauf des Arbeitsvertrages)	⓪	①	○
Berufliche Neuorientierung	⓪	①	○
Unzufriedenheit mit Organisation/Qualität der Weiter-/Facharztausbildung	⓪	①	○
Doppelbelastung durch Forschung und klinische Tätigkeit	⓪	①	○
Diskriminierung von Frauen	⓪	①	○
Andere Gründe (bitte nennen): ____			○

➥ **Bitte umblättern**

38.	Wenn Sie derzeit **klinisch tätig** sind, geben Sie bitte an, welches alternative Berufsfeld Sie sich am besten vorstellen können.<br>Wenn Sie derzeit **nicht-klinisch tätig** sind, geben Sie bitte an, in welchem alternativen Berufsfeld Sie derzeit tätig sind.<br>Bitte kreuzen Sie nur das Berufsfeld an, das **am ehesten** auf Sie zutrifft.		
☐ 0	Keines	☐ 4	Medizinmanagement
☐ 1	Lehre / Forschung an Fach(hoch)schulen oder Universitäten	☐ 5	Medizintechnik
☐ 2	Medien- oder Verlagsbranche	☐ 6	Pharmaindustrie
☐ 3	Medizininformatik	☐ 7	Gesundheitsverwaltung / -politik (Krankenkassen, Ministerien, etc.)
☐ 99	Sonstiges (bitte nennen, bitte **nicht** abkürzen):<br>____		

Wir danken Ihnen ganz herzlich für

das Ausfüllen des Fragebogens!
